# Destigmatising mental health treatment and increasing openness to seeking treatment: randomised controlled trial of brief video interventions

**DOI:** 10.1192/bjo.2022.575

**Published:** 2022-09-16

**Authors:** Doron Amsalem, Melanie Wall, Amit Lazarov, John C. Markowitz, Chana T. Fisch, Mariah LeBeau, Melissa Hinds, Jun Liu, Prudence W. Fisher, Thomas E. Smith, Sidney Hankerson, Roberto Lewis-Fernández, Yuval Neria, Lisa B. Dixon

**Affiliations:** Department of Psychiatry, New York State Psychiatric Institute, and Columbia University Vagelos College of Physicians & Surgeons, New York, NY, USA; School of Psychological Sciences, Tel Aviv University, Israel; Department of Psychiatry, New York State Psychiatric Institute, New York, NY, USA; Columbia University Vagelos College of Physicians & Surgeons, New York, and New York State Office of Mental Health, NY, USA; Department of Psychiatry, New York State Psychiatric Institute, Columbia University Vagelos College of Physicians & Surgeons, and Department of Epidemiology, Columbia University Irving Medical Center, New York, NY, USA

**Keywords:** Essential workers, COVID-19, openness to seeking treatment, stigma, intervention

## Abstract

**Background:**

Despite an elevated risk of psychopathology stemming from COVID-19-related stress, many essential workers stigmatise and avoid psychiatric care. This randomised controlled trial was designed to compare five versions of a social-contact-based brief video intervention for essential workers, differing by protagonist gender and race/ethnicity.

**Aims:**

We examined intervention efficacy on treatment-related stigma (‘stigma’) and openness to seeking treatment (‘openness’), especially among workers who had not received prior mental healthcare. We assessed effectiveness and whether viewer/protagonist demographic concordance heightened effectiveness.

**Method:**

Essential workers (*N* = 2734) randomly viewed a control video or brief video of an actor portraying an essential worker describing hardships, COVID-related anxiety and depression, and psychotherapy benefits. Five video versions (Black/Latinx/White and male/female) followed an identical 3 min script. Half the intervention group participants rewatched their video 14 days later. Stigma and openness were assessed at baseline, post-intervention, and at 14- and 30-day follow-ups. Trial registration: NCT04964570.

**Results:**

All video intervention groups reported immediately decreased stigma (*P* < 0.0001; Cohen's *d* = 0.10) and increased openness (*P* < 0.0001; *d* = 0.23). The initial increase in openness was largely maintained in the repeated-video group at day 14 (*P* < 0.0001; *d* = 0.18), particularly among viewers without history of psychiatric treatment (*P* < 0.0001; *d* = 0.32). Increases were not sustained at follow-up. Female participants viewing a female protagonist and Black participants viewing a Black protagonist demonstrated greater openness than other demographic pairings.

**Conclusions:**

Brief video-based interventions improved immediate stigma and openness. Greater effects among female and Black individuals viewing demographically matched protagonists emphasise the value of tailored interventions, especially for socially oppressed groups. This easily disseminated intervention may proactively increase care-seeking, encouraging treatment among workers in need. Future studies should examine intervention mechanisms and whether linking referrals to psychiatric services generates treatment-seeking.

Essential workers are employed in critical occupations considered indispensable for daily life (e.g. food service, transport and construction). According to recent research, essential workers have higher prevalence of anxiety and depressive disorders owing to the continuing stress of the COVID-19 pandemic.^1^ Increased workload, isolation from family and friends, emotional exhaustion and fear of viral transmission are among the reasons for increased psychopathology.^2,3^ Importantly, essential workers do not have the option to work from home and cannot afford to lose their jobs. Thus, they are exposed daily, sometimes unwillingly, to increased risk of infection.^4^ Moreover, Black and Latinx adults represent large proportions of essential workers in the USA; evidence suggests that members of underserved racial and ethnic groups have lower access to mental healthcare and often report more persistent and impairing depressive symptoms than non-Latinx individuals.^5,6^ These underserved groups are disproportionately affected by the pandemic, which raises their risk of mental health problems.^7–9^ Most research in this area has focused on healthcare workers; other essential workers have been relatively neglected.

There are many structural and other barriers to service use among essential workers and underserved racial and ethnic groups, including constraining work conditions and limited insurance.^10^ Another important obstacle is stigma towards mental healthcare.^11^ Treatment-related stigma involves perceiving mental-healthcare-seeking as weakness, anticipating negative attitudes from friends and families, and fearing discrimination from colleagues.^12,13^ Therefore, applying effective interventions to reduce treatment-related stigma and promote treatment-seeking may increase the likelihood that essential workers access care and may mitigate the risk of chronic, debilitating mental health problems.^14^

Previous research has shown that social-contact-based interventions are most effective in reducing stigma and increasing openness to seeking treatment.^15^ Social-contact-based interventions involve interaction with an empowered presenter with lived experience who describes coping successfully with distress and attaining their desired goals. Individuals interacting with these empowered presenters show decreased prejudice and discrimination related to mental health.^14,16^ Recent studies have reported that video-based social contact interventions have similar efficacy to in-person interventions in reducing stigma.^17,18^ Video-based interventions have the advantages of cost-effectiveness, scalability, replicability and ease of dissemination.^19^

Recently, our team demonstrated the efficacy of social-contact-based brief video interventions in increasing openness to seeking treatment for mental health conditions among US healthcare workers (*n* = 350)^20^ and military veterans (*n* = 172).^21^ In some cases, we found greater efficacy when the viewer and the protagonist shared sociodemographic characteristics such as gender, race/ethnicity and occupation, possibly by enhancing viewers’ identification and emotional engagement with the video protagonist.^20–22^ These exploratory studies were the first to employ such an intervention and demonstrate its effect on openness to seeking treatment. Nonetheless, they had limitations, which the present study aims to address.

First, the studies tested the intervention's efficacy only among healthcare workers and veterans, who differ from non-healthcare essential workers in having greater awareness of mental illness and greater access to care, respectively. To our knowledge, no previous study has examined the efficacy of such interventions among non-healthcare essential workers. Second, in each of the prior studies, we used a single video of a White woman or man, so we were unable to test the impact of gender or ethnoracial concordance between viewers and protagonists on the intervention effect among diverse groups of viewers. Third, both studies lacked data about treatment-related stigma and prior mental health treatment, which are crucial for understanding baseline perceptions and changes in openness to seeking treatment. Finally, the previous samples may have been underpowered to detect lasting interventional effects at 30-day follow-up or in specific subgroups, such as participants without history of mental health treatment.

We conducted a randomised controlled trial to test the efficacy of five versions of a brief video-based intervention, differing by protagonist gender and/or race/ethnicity, in reducing treatment-related stigma and increasing openness to seeking treatment in a larger sample (*n* = 2739) of US non-healthcare essential workers. Essential workers were randomly assigned to one of the video interventions or to a video control group. Assessments of treatment-related stigma and openness to seeking treatment were conducted at baseline, immediately post-intervention, and at 14- and 30-day follow-ups. Among the intervention groups, half of the participants viewed the same video again on day 14. We hypothesised that: (a) the brief video-based intervention would have the immediate and, on re-viewing, repeated effect of reducing treatment-related stigma and increasing openness to seeking treatment compared with the control condition; (b) viewing the video twice would be associated with greater durability of effect compared with viewing it once and with the control condition; (c) participants without history of treatment would experience greater intervention effects; and (d) viewer–protagonist concordance with respect to gender and/or race/ethnicity would yield greater effects on stigma reduction and openness to seeking treatment.

## Method

### Participants and recruitment

Participants were recruited through Prolific, a crowdsourcing tool frequently used in medical and psychology research with worldwide evidence of validity across tasks.^23^ Prolific ensures respondent consistency in demographic responses over time, blocks those who use tools to hide their location, runs checks to identify fake participants and creates anonymous respondent IDs. To further verify the validity and accuracy of the study's results, we excluded participants who answered the survey more than once and added a timer to ensure that respondents read the instructions (5 s minimum) and watched the video (170 s) before the ‘next’ button appeared. We also excluded respondents who failed our attention-testing questions (e.g. ‘In the following question, please choose the second answer’).

Recruitment took place during August and September of 2021. We only included essential workers who were aged 18–80 years, English-speaking and US residents. We defined ‘essential work’ as: (a) non-healthcare-related indispensable occupations (e.g. manufacturing, construction, transportation, food industry, hospitality and non-healthcare emergency services); (b) occupations that required daily travel to work during the COVID-19 pandemic. Participants were compensated $9 for study participation. The authors assert that all procedures contributing to this work comply with the ethical standards of the relevant national and institutional committees on human experimentation and with the Helsinki Declaration of 1975, as revised in 2008. All procedures involving human subjects/patients were approved by the New York State Psychiatric Institute Institutional Review Board (#8128). Before study entry, participants reviewed an informed consent form. Those agreeing to participate completed the study procedures via Qualtrics.com, a secure, online data-collection platform.

### Procedure

Participants were randomised to either video intervention or video control groups. The video intervention group included five brief video versions, which varied by the race/ethnicity and gender of the protagonist – Black female, Latinx female, Latinx male, White female and White male. Participants were evenly divided between the Black, Latinx and White protagonists. The control group watched a same-length video featuring nature views and horses, accompanied by relaxing music. The pre-intervention survey included demographic and COVID-19-related information, and questionnaires assessing treatment-related stigma and openness to seeking treatment. The post-intervention survey, conducted immediately after the intervention, included the same assessments of treatment-related stigma and openness to seeking treatment as the baseline survey. Fourteen days later, half of the participants in the intervention groups rewatched the same video (‘repeated video’), whereas the remaining participants had no additional intervention (‘video’). Follow-ups included assessment of treatment-related stigma and openness to seeking treatment and were conducted at 14 and 30 days post-intervention (see CONSORT checklist in the supplementary material, available at https://doi.org/10.1192/bjo.2022.575).

### Intervention

The intervention included five brief videos, each roughly 3 min long, that presented an identical, scripted story of an essential worker. The only difference across videos was the protagonist's race/ethnicity and gender. Protagonists described their difficulty coping with life stressors, how they experienced symptoms of anxiety and depression during the pandemic, and prior misconceptions about treatment, and how they overcame them. The protagonists described benefiting from social support and mental health therapy and how it helped them cope with their stressors. They all concluded with a supportive, encouraging statement: ‘I have become calmer, more in control. I started to enjoy the little things and become myself again. I really wish I would have spoken with someone sooner’. The five videos are available through video links in Supplementary Appendix 1.

### Instruments

We measured treatment-related stigma with the Self-Stigma of Seeking Help (SSOSH-3) scale.^24^ The three items were: ‘It would make me feel inferior to ask a therapist for help’, ‘I would feel inadequate if I went to a therapist for psychological help’, and ‘If I went to a therapist, I would be less satisfied with myself’. Response choices ranged from 1 (strongly disagree) to 5 (strongly agree). Total scores ranged from 3 to 15, with lower scores indicating less stigma. In this study, Cronbach's alpha was 0.87.

Openness to seeking treatment was determined by the three ‘openness to help-seeking’ items from the Attitude Towards Seeking Professional Psychological Help (ATSPPH) scale – Short Form.^25^ The items were: ‘I would want to get psychological help if I were worried or upset for a long period of time’, ‘I might want to have psychological counseling in the future’ and ‘A person with an emotional problem is not likely to solve it alone; they are more likely to solve it with professional help’. Responses ranged from 1 (disagree) to 4 (agree), for a total score of 3 to 12, with higher scores indicating greater openness to seeking treatment. In this study, Cronbach's alpha was 0.83.

### Data analysis

Pearson's χ² and one-way analysis of variance (ANOVA) were used to compare sociodemographic and COVID-19-related characteristics across the five versions of the video intervention group and the control group, and independent *t*-tests were used to compare SSOSH (stigma) and ATSPPH (openness to treatment) scores between video intervention and control groups. Intervention effects were examined using generalised estimating equations (GEE),^26^ as recommended for randomised controlled trials.^27^ The GEE approach represents correlated repeated-measures analysis and calculates missing data via estimated marginal means based on the whole sample. It includes all randomised participants providing data for at least at one time point. To account for within-respondent dependencies in the models, we specified an unstructured correlation matrix. We applied a full factorial model across the four time points (baseline, immediately post-intervention, and 14 day and 30 day follow-ups) for the SSOSH and ATSPPH. To test our first hypothesis, we examined the immediate and repeated effects (video versus control) with time-by-group interaction terms. Using the GEE model, we tested the 14-day video effects (single video versus repeated-video versus control), addressing our second hypothesis about durability of effects. For the third hypothesis, we first conducted a one-way ANOVA examining whether baseline SSOSH and ATSPPH mean scores of each participant subgroup (reporting past therapy, current therapy or no prior therapy) significantly differed. We then conducted the same GEE analysis separately for each subgroup. For the fourth hypothesis, we tested whether viewer–protagonist concordance in gender and/or race/ethnicity yielded a greater effect among respondents (i.e. female viewers watching female protagonists versus females watching males, or Black viewers watching a Black protagonist versus Black viewers watching White or Latinx protagonists). We conducted two sets of analyses, first addressing concordance by gender and then concordance by race/ethnicity. The sample size was not large enough to address the intersectionality of gender and race/ethnicity simultaneously (e.g. Black female watching a Black female protagonist). To test the gender-concordance hypothesis, we expanded the GEE model to include fixed effects for the participant's and protagonist's genders and their interaction and the full factorial interaction of these with video condition and time; this allowed us to test for differential effects for each participant's gender as a function of protagonist gender immediately following the video or the repeated video, and any lasting effects. Contrasts measuring the concordance effect were formed specifically for gender and overall; that is, we obtained a concordance effect specifically for women separately from a concordance effect for men (gender-specific concordance) and we formed an overall gender-concordance effect (i.e. females watching females aggregated with males watching males) contrasting with overall gender discordance (females watching males plus males watching females). Participants who indicated their gender to be other than male or female were excluded from this analysis owing to small numbers. Similar analyses were conducted to test the concordance hypothesis by race/ethnicity, including a fixed effect for participant and actor race/ethnicity. These analyses were limited to participants who self-identified as Black, Latinx, or White so that concordance was possible, as the actors were limited to these races/ethnicities. In the race/ethnicity-concordance analysis, we also included the gender of the participant as a control variable to account for any confounding imbalance due to there being only a female Black actor (no male Black actor) condition. Finally, we ran all concordance analyses restricted to the subsample that had not previously received mental health treatment as we expected this group would have the largest effect sizes. Effect sizes are reported using Cohen's *d*, when appropriate, obtained by scaling mean differences between treatment groups by the standard deviation of the measure across all groups at baseline. All statistical tests were two-sided, using α < 0.05.

We collected data on clinical symptoms including level of self-reported anxiety and depression. We found no association between symptom severity and intervention effect on stigma or treatment-seeking response.

## Results

### Sample characteristics

**Fig. 1:**
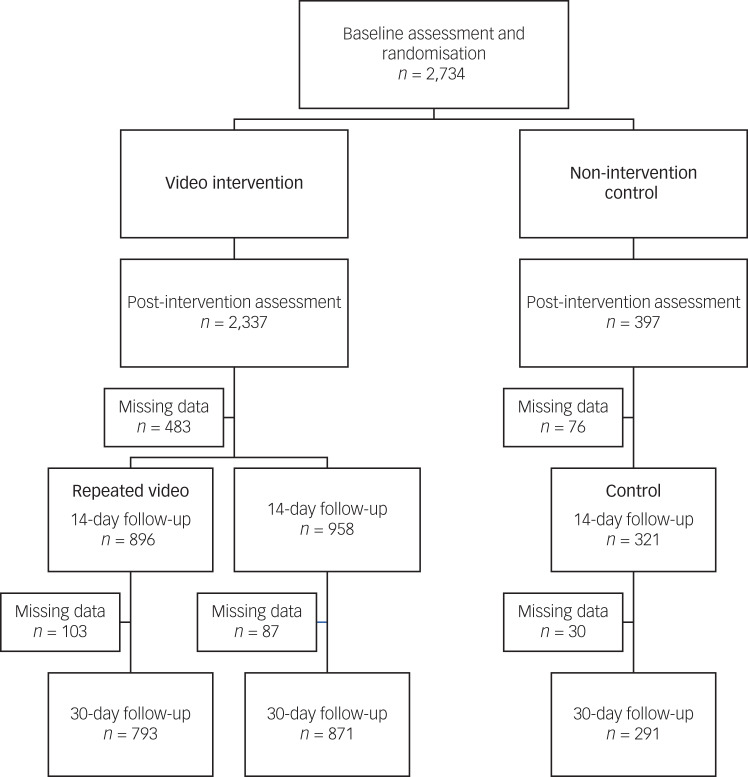
Study profile (August–September 2021).

**Fig. 2:**
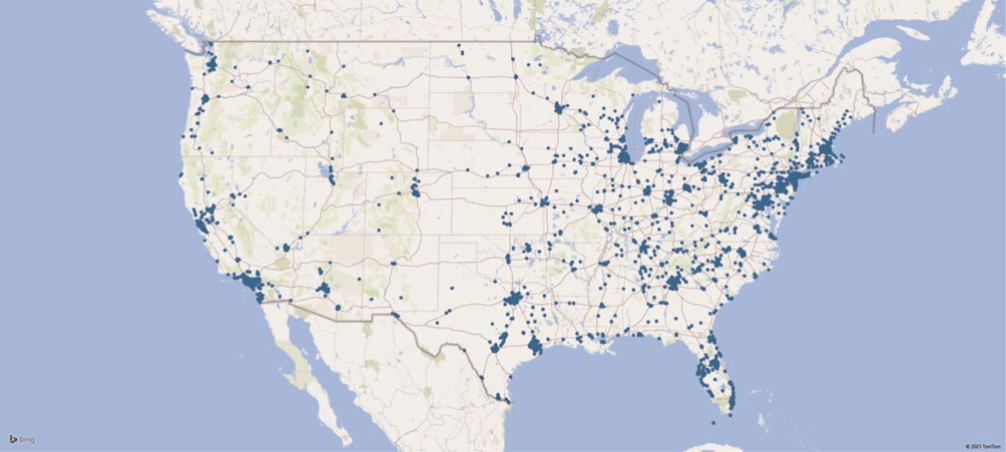
Essential workers’ geographic distribution (August–September 2021).

Of the recruited sample, 195 (7%) individuals failed validity tests and were excluded from analyses. The final sample included 2374 essential workers (62% female, *M*_age_ = 27.1 ± 9.3 years, range 18–73) who completed the baseline and post-intervention assessments. Of those, 80% (*n* = 2175) completed the 14-day assessment and 72% (*n* = 1955) completed the 30-day assessment (Fig. 1). Groups did not differ on demographic and COVID-19-related characteristics. Baseline characteristics did not differ between study completers and non-completers. Table 1 presents the race and ethnicity, essential occupations and COVID-19-related characteristics of participants. Figure 2 illustrates participants’ geographic distribution.

**Table 1:** Demographic and COVID-19-related characteristics, *n* (%)

Items	Black woman video *n* = 780	Hispanic woman video *n* = 388	Hispanic man video *n* = 392	White woman video *n* = 392	White man video *n* = 385	Control video *n* = 397	Total *n* = 2734	Statistic	*P*-value
Mean age, years (s.d.)	27.4 ± 9.8	27.1 ± 9.1	26.7 ± 9.0	26.7 ± 8.4	27.1 ± 9.5	27.0 ± 9.2	27.1 ± 9.3	0.42^a^	0.83
Gender – female	496 (63)	237 (61)	244 (62)	242 (62)	241 (63)	242 (61)	1,702 (62)	1.2^b^	0.95
Race and ethnicity								13.2^b^	0.97
Hispanic/Latino	109 (14)	57 (15)	57 (15)	56 (14)	57 (15)	58 (15)	394 (14)		
Non-Hispanic White	521 (67)	255 (66)	260 (66)	257 (66)	246 (64)	262 (66)	1801 (66)		
Non-Hispanic Black	72 (9)	35 (9)	34 (9)	40 (10)	34 (9)	37 (9)	252 (9)		
Non-Hispanic Asian	46 (6)	27 (7)	24 (6)	24 (6)	38 (10)	26 (7)	185 (7)		
Non-Hispanic Native American/Pacific Islander	7 (1)	2 (1)	6 (2)	3 (1)	2 (1)	2 (1)	22 (1)		
Non-Hispanic Other^c^	25 (3)	12 (3)	11(3)	12(3)	68(2)	12 (3)	80 (3)		
Occupation								36.5^b^	0.81
Food industry	231 (30)	117 (30)	120 (31)	99 (25)	120 (31)	132 (33)	819 (30)		
Retail/sales	144 (18)	75 (19)	84 (21)	76 (19)	66 (17)	67 (17)	512 (19)		
Manufacturing	124 (16)	56 (14)	55 (14)	65 (17)	59 (15)	67 (17)	426 (16)		
Construction	64 (8)	40 (10)	38 (10)	33 (8)	29 (8)	28 (7)	232 (8)		
Transportation	40 (5)	14 (4)	18 (5)	26 (7)	22 (6)	23 (6)	143 (5)		
Education	42 (5)	15 (4)	14 (4)	26 (7)	26 (7)	19 (5)	142 (5)		
Non-healthcare emergency services	36 (5)	24 (6)	19 (5)	19 (5)	17 (5)	20 (5)	135 (5)		
Veterinary and animal care	14 (2)	8 (2)	13 (3)	5 (1)	6 (2)	7 (2)	53 (2)		
Hospitality	9 (1)	7 (2)	3 (1)	6 (2)	5 (1)	6 (2)	36 (1)		
Other	76 (10)	32 (8)	28 (7)	37 (9)	35 (9)	28 (7)	236 (9)		
Tested positive for COVID-19	132 (17)	59 (15)	62 (16)	71 (18)	66 (17)	59 (15)	449 (16)	4.3^b^	0.93
Family/friend tested positive for COVID-19	569 (73)	295 (76)	293 (75)	276 (70)	271 (70)	270 (68)	1,974 (72)	11.5^b^	0.32
Exposed to sick people at work	426 (55)	206 (53)	214 (55)	225 (57)	224 (58)	226 (57)	1,521 (56)	6.4^b^	0.78
Received the COVID-19 vaccine	605 (78)	296 (76)	318 (81)	288 (74)	304 (79)	319 (80)	2,130 (78)	10.8^b^	0.37
Sought psychological counselling	382 (49)	200 (52)	193 (49)	183 (47)	163 (42)	170 (43)	1,291 (47)	15.8^b^	0.10

a. One-way ANOVA.

b. Pearson chi-squared.

c. Non-Hispanic Other: multiracial (*n* = 50), Middle Eastern (*n* = 2), unspecified (*n* = 28).

### Intervention effects

#### Stigma scores for full group

**Fig. 3:**
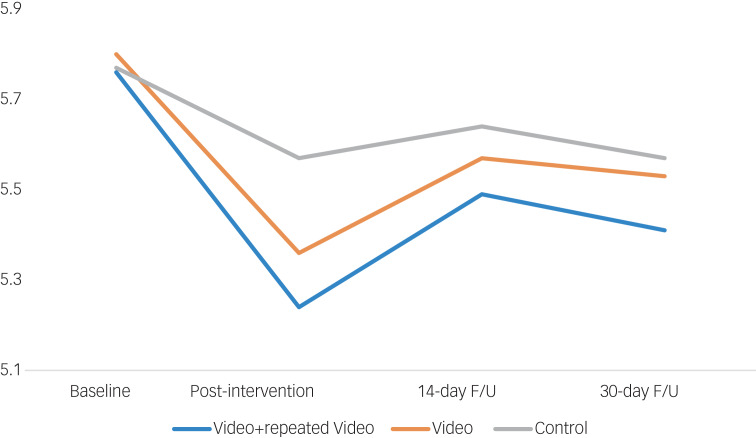
Immediate, repeated and longer-term effects on Self-Stigma of Seeking Help scale. Total scores ranged from 3 to 15, with higher scores indicating greater stigma (3 = disagree; 6 = partly disagree; 9 = neutral; 12 = partly agree; 15 = agree); F/U, follow-up.

Figure 3 presents the GEE model results for stigma (SSOSH) scores. Groups did not differ significantly on baseline mean scores (video: 5.8 [95% CI: 5.7, 5.9]; control: 5.8 [5.5, 6.1]; independent *t*-test: *t* = 0.08, *P* = 0.94). A significant group-by-time interaction emerged (x^2^ = 24.0, d.f. = 1, *P* < 0.0001) for an immediate effect (baseline to post-intervention change: 0.28 [0.17, 0.39]; Cohen's *d* = 0.10). We found no group-by-time interaction for the repeated-video effect, nor any lasting effects at 14-day or 30-day follow-up for the intervention groups.

### Openness to seeking treatment scores for full group

**Fig. 4:**
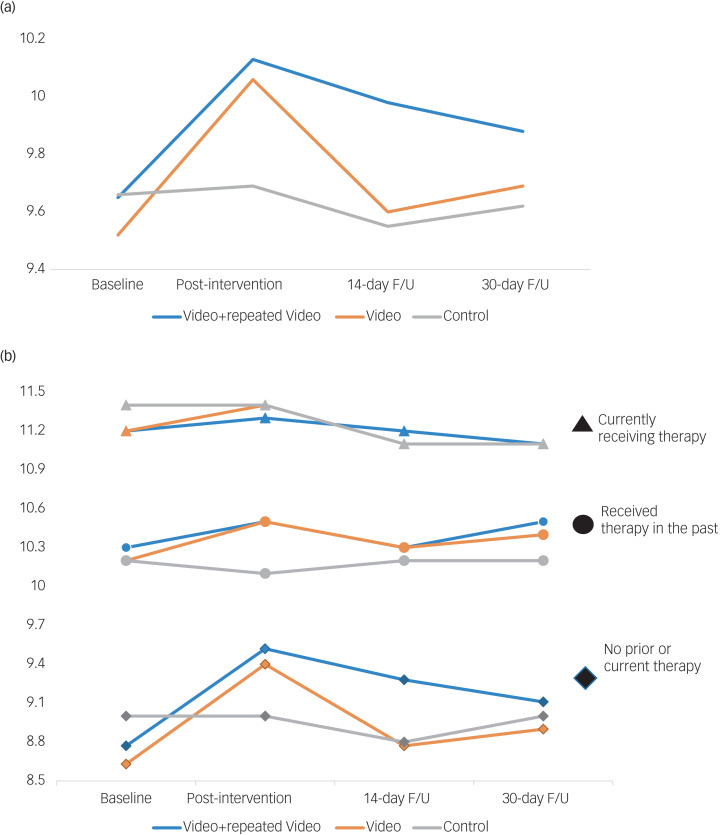
(a) Immediate, repeated and longer-term effects on Attitudes Towards Seeking Professional Psychological Help (ATSPPH) scale. (b) Immediate, repeated and longer-term effects on ATSPPH divided by the response to ‘have you sought psychological counseling?’. Total scores ranged from 3 to 12, with higher scores indicating greater openness to seeking treatment (3 = disagree; 6 = partly disagree; 7.5 = neutral; 9 = partly agree; 12 = agree); F/U, follow-up.

Figure 4(a) presents the GEE model results for openness to seeking treatment. The groups did not differ on baseline mean ATSPPH scores (video groups: 9.6 [9.5, 9.7]; control group: 9.7 [9.4, 9.9]; independent *t*-test: *t* = 0.70, *P* = 0.48). Analyses showed a group-by-time interaction (*x*^2^ = 74.7, d.f. = 1, *P* < 0.0001; Cohen's *d* = 0.23) for an immediate effect (baseline to post-intervention change: 0.48 [0.37, 0.59]) and a group-by-time interaction (*x*^2^ = 18.4, d.f. = 1, *P* < 0.0001; Cohen's *d* = 0.18) for the repeated-video effect (baseline to 14-day follow-up change: 0.38 [0.20, 0.55]). We found no significant difference between the magnitude of immediate and repeated-video effects (*x*^2^ = 1.5, d.f. = 1, *P* = 0.22). There was no lasting effect at 14-day follow-up for the single-video or at 30-day follow-up for the repeated-video groups. Hence, repeating the video led to no significantly greater durability of effect.

### Openness to seeking treatment scores by therapy status

We repeated the above-described GEE analysis separately based on treatment status in line with participants’ answers to ‘Have you sought psychological counseling?’, with answers/groups being ‘no prior therapy’, ‘past therapy’, or ‘current therapy’ (Fig. 4(b)). Baseline ATSPPH scores differed significantly across the three groups: no prior therapy: 8.7, [8.6, 8.9]; past therapy: 10.2 [10.1, 10.3]; current therapy: 11.2 [11.1, 11.3]; one-way ANOVA: F = 224.4, *P* < 0.001. Participants without prior therapy showed a group-by-time interaction (*x*^2^ = 65.3, *P* < 0.0001; Cohen's *d* = 0.32) for an immediate effect (baseline to post-intervention change: 0.69 [0.52, 0.85]) and a group-by-time interaction (*x*^2^ = 29.2, d.f. = 1, *P* < 0.0001; Cohen's *d* = 0.33) for a repeated-video effect (baseline to 14-day follow-up change: 0.70 [0.44, 0.95]). Participants reporting therapy in the past but not currently showed a group-by-time interaction (*x*^2^ = 15.0, d.f. = 1, *P* < 0.001; Cohen's *d* = 0.16) for the immediate-video effect only (baseline to post-intervention change: 0.35 [0.17, 0.53]). Participants who reported currently receiving therapy already had near-maximal scores and showed no immediate intervention effects. There was no lasting effect at 14-day follow-up for the single-video or at 30-day follow-up for the repeated-video for any subgroup.

### Stigma and openness to seeking treatment scores by concordance in gender or race/ethnicity

In the full sample, protagonist gender or race/ethnicity did not influence changes in outcome of stigma and/or openness to seeking treatment. However, among respondents with no prior treatment, concordance between the viewer's and protagonist's gender and/or race/ethnicity yielded a greater effect in some groups (Supplementary Appendix 2 and 3). For example, regarding treatment-related stigma, overall gender concordance (i.e. female-watching-female plus male-watching-male) yielded a greater immediate decrease in stigma scores. Females watching female protagonists showed greater decreases in SSOSH scores than females watching male protagonists (concordant – baseline to post-intervention change: 0.40 [0.10], *P* < 0.0001, Cohen's *d* = 0.15; discordant – baseline to post-intervention change: 0.21 [0.12], *P* = 0.07, Cohen's *d* = 0.08). Males watching male protagonists showed greater decreases in SSOSH scores than males watching female protagonists (0.38 [0.15], *P* = 0.01, Cohen's *d* = 0.14 *v*. 0.16 [0.14], *P* = 0.25, Cohen's *d* = 0.06); between-group change (concordant *v*. discordant): 0.22 [0.19], *P* = 0.009, Cohen's *d* = 0.08.

Regarding openness to seeking treatment, females watching female protagonists showed greater increases in ATSPPH scores than females watching male protagonists (0.75 [0.12], *P* < 0.0001, Cohen's *d* = 0.35 *v*. 0.49 [0.13], *P* < 0.0001, Cohen's *d* = 0.23; between-group change: 0.26 [0.10], *P* = 0.01, Cohen's *d* = 0.12). However, males watching male protagonists did not show any greater increase in ATSPPH scores than males watching female protagonists (0.66 [0.16], *P* < 0.0001, Cohen's *d* = 0.31 *v*. 0.75 [0.14], *P* < 0.0001, Cohen's *d* = 0.35; between-group change: –0.09 [0.10], *P* = 0.50).

Regarding ethnoracial concordance, no concordance effect was found for treatment-related stigma (SSOSH). For openness to seeking treatment, Black participants viewing Black protagonists showed greater immediate increases in ATSPPH scores than Black participants viewing non-Black protagonists (1.57 [0.44], *P* < 0.001, Cohen's *d* = 0.73 *v*. 0.72 [0.37], *P* = 0.05, Cohen's *d* = 0.34; between-group change: 0.85 [0.35], *P* = 0.01, Cohen's *d* = 0.39). We found a similar pattern for the repeated-video effect (1.63 [0.65], *P* = 0.01, Cohen's *d* = 0.76 *v*. 0.78 [0.63], *P* = 0.21, Cohen's *d* = 0.36; between-group change: 0.85 [0.35], *P* = 0.04, Cohen's *d* = 0.42). No concordance effect was observed for Latinx–Latinx or White–White pairings.

## Discussion

Our randomised controlled trial tested the efficacy of five versions of a brief video intervention differing by protagonist gender and/or race/ethnicity in reducing treatment-related stigma and increasing openness to seeking treatment among 2734 essential workers. In brief (3 min) videos, essential workers described their emotional struggles during the COVID-19 pandemic and how mental health treatment helped them cope. As hypothesised, viewing any of the five versions of the video intervention led to immediate decreases in treatment-related stigma and increases in openness to seeking treatment compared with the control condition. The initial increase in openness to seeking treatment was largely maintained in the repeated-video group at day 14. These findings replicate our previous exploratory studies among healthcare workers^20^ and veterans.^21^ To our knowledge, our study is the first to demonstrate the efficacy of a social-contact-based intervention in reducing treatment-related stigma and increasing openness to seeking treatment among the understudied population of non-healthcare essential workers.

Among participants without prior treatment, female or Black viewers watching female or Black protagonists demonstrated a greater immediate increase in openness to seeking treatment than those watching male or non-Black protagonists. These findings are consistent with our previous studies with a single protagonist, which showed secondary findings that may imply a concordance effect. For example, in a study about psychosis-related stigma, we demonstrated greater stigma reduction among females watching a female protagonist (versus males watching a female protagonist)^22^ and a greater increase in openness to seeking treatment among male veterans watching a male protagonist (versus females watching a male protagonist).^21^ Nonetheless, this is the first study to compare several versions of the same video differing in protagonist gender and/or race/ethnicity, thus enabling a test of the concordance hypothesis. Similarly, a study on patient–clinician communication showed higher patient ratings for satisfaction and participation in decision-making among race-concordant groups.^28^ However, few studies have examined cultural aspects of stigma-reduction interventions^10^ and, to our knowledge, no other studies on social-contact-based interventions have examined such an effect.

Why were our concordance effect findings restricted only to female and Black participants, whereas responses of male or White participants did not vary by protagonist gender and/or race/ethnicity? One explanation lies in the core differences within each category: female and Black individuals are more likely to be socially oppressed than male and White individuals. This underscores the importance of matching viewer and protagonist sociodemographics, especially among members of marginalised groups who are more aware of discrimination and therefore may place greater value on solidarity and identification. However, we did not find similar effects among Latinx viewers. Past studies have shown that mental health stigma is higher among Spanish-speaking Latinx individuals, who also have lower perceptions of need of mental healthcare,^29^ whereas our sample included English-speaking Latinx individuals only. Perhaps we would find a greater concordance effect among Spanish-speaking Latinx people watching Spanish-speaking protagonists. Another explanation may be related to lack of power to detect such an effect. In addition to increased sample sizes, future studies should explore the mechanisms of action of the ethnoracial concordance and whether content adjusted to explicitly address these sociodemographic differences creates even greater effects, for example, a Black protagonist describing how their racial background imposes greater difficulties in accessing mental healthcare.

This study replicates and extends our previous pilot studies in several important ways. First, its larger sample size (*n* = 2734) allowed us to study this underserved group that has been disproportionally affected by the COVID-19 pandemic and faces an increased risk of mental health problems.^7–9,30^ This is the first study to show such an effect among non-healthcare essential workers. Second, we assessed intervention effects on stigma, a key deterrent to treatment-seeking,^11^ showing an immediate effect not only in increasing openness to seeking treatment but also in decreasing treatment-related stigma. We found a greater effect among respondents who had never sought or received treatment (53% of the sample), thus strengthening the intervention effect and emphasising its necessity within this in-need subgroup. Last, we tested five versions of the video intervention, altering the gender and race/ethnicity of the protagonist, thus increasing both external and internal validity.

Although results showed that the intervention had an immediate impact, no lasting effect emerged: effects did not persist at 14 day follow-up after a single video, or at 30 day follow-up following the repeated video. These findings corroborate those of other intervention studies^31^ among healthcare workers, mainly using non-contact-based educational programmes, which also showed no lasting effects. Perhaps, instead of expecting longer durability of a brief video effect, the goal should be to augment the immediate effect on openness to seeking treatment and focus on how this can be leveraged to create behaviour change. The short-term increase in openness to seeking treatment, which our follow-up data show can be repeated, creates a time-limited window of opportunity to connect essential workers with mental health services. Short video marketing research^32^ shows that video platforms that introduce an actionable function to focus the desired intention change (e.g. a linkable shopping cart on commercial advertisements) have significantly increased efficacy compared with identical videos lacking this function. For healthcare access, adding a referral to crisis counsellors or an option to connect with a therapist might engage in-need essential workers with mental health services.

Treatment-related stigma and treatment-seeking perceptions are not the only barriers to care for essential workers. Structural and other barriers to healthcare significantly impede access to timely care.^33^ Such issues are longstanding and are likely to remain well beyond this pandemic. Telehealth and digital solutions can be key strategies in linking mental health services to essential workers.^34,35^ For example, interventions informed by cognitive–behavioural therapy, delivered by mobile apps, may help address infrastructure barriers to accessing care.^36,37^ However, other studies show limited efficacy for such treatments.^38^

### Limitations

This study had several limitations. First, the users of crowdsourcing platforms may differ sociodemographically from the essential worker population, limiting generalisability. Ethnoracially, however, our sample resembled the 2020 US census distribution fairly closely: 14% (study) *v*. 16% (US) Hispanic/Latino, 66% *v*. 64% non-Hispanic White, 10% *v*. 12% non-Hispanic Black and 7% *v*. 5% non-Hispanic Asian. Second, we did not assess a video featuring a Black male protagonist. Future studies should explore a wider range of gender and race/ethnicity, with sufficiently large samples to study their intersections. Third, we assessed openness to seeking treatment, a measure possibly influenced by social desirability.^39^ Unfortunately, no research to date, including our own, has measured effects on actual treatment-seeking behaviour. Last, our study may have lacked the power to detect concordance effects among Latinx and male groups. Future studies should explore whether greater tailoring creates a larger effect.

In summary, this randomised controlled trial replicated and extended our previous findings, showing positive effects of five versions of a social-contact-based brief video intervention, differing with respect to protagonist gender and/or race/ethnicity, especially among essential workers who had received no prior treatment. The 3 min videos showed a modest effect size of reduced treatment-related stigma and increased immediate openness to seeking treatment, with greater effects among female and Black viewers who watched protagonists with matching sociodemographic characteristics. Policy makers and employee assistance programmes should consider using such easily disseminatable interventions to proactively encourage essential workers to seek help and to provide mental health services to those needing them. Future studies should examine the mechanisms of action of these brief video interventions and whether links to referrals could foster immediate behavioural change.

## Data Availability

The data that support the findings of this study are available from the corresponding author on reasonable request.

## Supplementary material

For supplementary material accompanying this paper visit http://doi.org/10.1192/bjo.2022.575. click here to view supplementary material

## References

[1] De Boni RB, Balanzá-Martínez V, Mota JC, Cardoso TA, Ballester P, Atienza-Carbonell B, Depression, anxiety, and lifestyle among essential workers: a web survey from Brazil and Spain during the COVID-19 pandemic. J Med Internet Res 2020; 22(10): e22835. 33038075 10.2196/22835 PMC7641648

[2] Bell C, Williman J, Beaglehole B, Stanley J, Jenkins M, Gendall P, Challenges facing essential workers: a cross-sectional survey of the subjective mental health and well-being of New Zealand healthcare and other essential workers during the COVID-19 lockdown. BMJ Open 2021; 11(7): e048107. 10.1136/bmjopen-2020-048107 PMC8290948 34281926

[3] Fang X, Zhang J, Teng C, Depressive symptoms in the front-line non-medical workers during the COVID-19 outbreak in Wuhan. J Affect Disord 2020; 276: 441–5. 32871675 10.1016/j.jad.2020.06.078 PMC7365080

[4] Mazza M, Attanasio M, Pino MC, Moral decision-making, stress, and social cognition in frontline workers vs. population groups during the COVID-19 pandemic: an explorative study. Front Psychol 2020; 11: 588159. 33329249 10.3389/fpsyg.2020.588159 PMC7710972

[5] Youman K, Drapalski A, Stuewig J, Bagley K, Tangney J. Race differences in psychopathology and disparities in treatment seeking: community and jail-based treatment-seeking patterns. Psychol Serv 2010; 7(1): 11. 21814487 10.1037/a0017864 PMC3148100

[6] Gawthrop E. The Color of Coronavirus: COVID-19 Deaths by Race and Ethnicity in the U.S. APM Research Lab: American Public Media Group, 2022 (https://www.apmresearchlab.org/covid/deaths-by-race [cited 5 Sep 2022]).

[7] Lancet T. The plight of essential workers during the COVID-19 pandemic. Lancet 2020; 395(10237): 1587. 32446399 10.1016/S0140-6736(20)31200-9 PMC7241973

[8] Tai DBG, Shah A, Doubeni CA, Sia IG, Wieland ML. The disproportionate impact of COVID-19 on racial and ethnic minorities in the United States. Clin Infect Dis 2021; 72(4): 703–6. 32562416 10.1093/cid/ciaa815 PMC7337626

[9] Stringer SM. New York City’s Frontline Workers. Bureau of Policy & Research: City of New York – Office of the Comptroller, 2020 (https://comptroller.nyc.gov/reports/new-york-citys-frontline-workers/ [cited 5 Sep 2022]).

[10] Misra S, Jackson VW, Chong J, Choe K, Tay C, Wong J, Systematic review of cultural aspects of stigma and mental illness among racial and ethnic minority groups in the United States: implications for interventions. Am J Community Psychol 2021; 68(3–4): 486–512. 33811676 10.1002/ajcp.12516

[11] Clement S, Schauman O, Graham T, What is the impact of mental health-related stigma on help-seeking? A systematic review of quantitative and qualitative studies. Psychol Med 2015; 45(1): 11–27. 24569086 10.1017/S0033291714000129

[12] Smith JR, Workneh A, Yaya S. Barriers and facilitators to help-seeking for individuals with posttraumatic stress disorder: a systematic review. J Trauma Stress 2020; 33(2): 137–50. 31697863 10.1002/jts.22456

[13] Center C, Davis M, Detre T, Confronting depression and suicide in physicians: a consensus statement. J Am Med Assoc 2003; 289(23): 3161–6. 10.1001/jama.289.23.3161 12813122

[14] Rüsch N, Angermeyer MC, Corrigan PW. Mental illness stigma: concepts, consequences and initiatives to reduce stigma. Eur Psychiatry 2005; 20(8): 529–39. 16171984 10.1016/j.eurpsy.2005.04.004

[15] Thornicroft G, Mehta N, Clement S, Evidence for effective interventions to reduce mental-health-related stigma and discrimination. Lancet 2016; 387(10023): 1123–32. 26410341 10.1016/S0140-6736(15)00298-6

[16] Corrigan PW, Michaels PJ, Vega E, Gause M, Larson J, Krzyzanowski R, Key ingredients to contact-based stigma change: a cross-validation. Psychiatr Rehabil J 2014; 37(1): 62–4. 24417232 10.1037/prj0000038

[17] Janoušková M, Tušková E, Weissová A, Can video interventions be used to effectively destigmatize mental illness among young people? A systematic review. Eur Psychiatry 2017; 41: 1–9. 28049074 10.1016/j.eurpsy.2016.09.008

[18] Koike S, Yamaguchi S, Ojio Y, A randomised controlled trial of repeated filmed social contact on reducing mental illness-related stigma in young adults. Epidemiol Psychiatr Sci 2018; 27(2): 199–208. 27989255 10.1017/S2045796016001050 PMC7032789

[19] Amsalem D, Dixon LB, Neria Y. The coronavirus disease 2019 (COVID-19) outbreak and mental health: current risks and recommended actions. JAMA Psychiatry 2021; 78(1): 9–10. 32579160 10.1001/jamapsychiatry.2020.1730

[20] Amsalem D, Lazarov A, Markowitz JC, Smith TE, Dixon LB, Neria Y. Video intervention to increase treatment-seeking by healthcare workers during the COVID-19 pandemic: randomised controlled trial. Br J Psychiatry 2022; 220(1): 14–20. 35045900 10.1192/bjp.2021.54

[21] Amsalem D, Lazarov A, Markowitz JC, Gorman D, Dixon LB, Neria Y. Increasing treatment-seeking intentions of US veterans in the Covid-19 era: a randomized controlled trial. Depress Anxiety 2021; 38(6): 639–47. 33734539 10.1002/da.23149 PMC8251313

[22] Amsalem D, Yang LH, Jankowski S, Lieff SA, Markowitz JC, Dixon LB. Reducing stigma toward individuals with schizophrenia using a brief video: a randomized controlled trial of young adults. Schizophr Bull 2021; 47(1): 7–14. 10.1093/schbul/sbaa114 PMC7825082 33484269

[23] Peer E, Brandimarte L, Samat S, Acquisti A. Beyond the Turk: alternative platforms for crowdsourcing behavioral research. J Exp Soc Psychol 2017; 70: 153–63.

[24] Brenner RE, Colvin KF, Hammer JH, Vogel DL. Using item response theory to develop revised (SSOSH-7) and ultra-brief (SSOSH-3) Self-Stigma of Seeking Help scales. Assessment 2021; 28(5): 1488–99. 32975438 10.1177/1073191120958496

[25] Elhai JD, Schweinle W, Anderson SM. Reliability and validity of the attitudes toward seeking professional psychological help scale-short form. Psychiatry Res 2008; 159(3): 320–9. 18433879 10.1016/j.psychres.2007.04.020

[26] Zeger SL, Liang K-Y, Albert PS. Models for longitudinal data: a generalized estimating equation approach. Biometrics 1988; 44: 1049–60. 3233245

[27] Vens M, Ziegler A. Generalized estimating equations and regression diagnostics for longitudinal controlled clinical trials: a case study. Comput Stat Data Anal 2012; 56: 1231–42.

[28] Cooper LA, Roter DL, Johnson RL, Ford DE, Steinwachs DM, Powe NR. Patient-centered communication, ratings of care, and concordance of patient and physician race. Ann Intern Med 2003; 139(11): 907–15. 14644893 10.7326/0003-4819-139-11-200312020-00009

[29] Breslau J, Cefalu M, Wong EC, Burnam MA, Hunter GP, Florez KR, Racial/ethnic differences in perception of need for mental health treatment in a US national sample. Soc Psychiatry Psychiatr Epidemiol 2017; 52(8): 929–37. 28550518 10.1007/s00127-017-1400-2 PMC5534379

[30] Smith K, Bhui K, Cipriani A. COVID-19, mental health and ethnic minorities. Evid Based Ment Health 2020; 23(3): 89–90. 32680834 10.1136/ebmental-2020-300174 PMC7418618

[31] Maunder RG, Lancee WJ, Mae R, Computer-assisted resilience training to prepare healthcare workers for pandemic influenza: a randomized trial of the optimal dose of training. BMC Health Serv Res 2010; 10: 72. 20307302 10.1186/1472-6963-10-72 PMC2851711

[32] Xiao Y, Wang L, Wang P. Research on the influence of content features of short video marketing on consumer purchase intentions. In *International Conference on Modern Management, Education Technology and Social Science (MMETSS 2019)*: 415–22. Atlantis Press, 2019.

[33] Becot F, Inwood S, Bendixsen C, Henning-Smith C. Health care and health insurance access for farm families in the United States during COVID-19: essential workers without essential resources? J Agromedicine 2020; 25(4): 374–7. 32921286 10.1080/1059924X.2020.1814924 PMC11075044

[34] Torous J, Andersson G, Bertagnoli A, Christensen H, Cuijpers P, Firth J, Towards a consensus around standards for smartphone apps and digital mental health. World Psychiatry 2019; 18(1): 97. 30600619 10.1002/wps.20592 PMC6313231

[35] Mata-Greve F, Johnson M, Pullmann MD, Friedman EC, Fillipo IG, Comtois KA, Mental health and the perceived usability of digital mental health tools among essential workers and people unemployed due to COVID-19: cross-sectional survey study. JMIR Ment Health 2021; 8(8): e28360. 34081592 10.2196/28360 PMC8354319

[36] Benhamou K, Piedra A. CBT-informed interventions for essential workers during the COVID-19 pandemic. J Contemp Psychother 2020; 50(4): 275–83. 32836379 10.1007/s10879-020-09467-3 PMC7367784

[37] Rathbone AL, Prescott J. The use of mobile apps and SMS messaging as physical and mental health interventions: systematic review. J Med Internet Res 2017; 19(8): e295. 28838887 10.2196/jmir.7740 PMC5590007

[38] Markowitz JC, Milrod BL. Postpandemic psychotherapy: still under siege. Psychiatr Ser 2022; 73(6): 690–2. 10.1176/appi.ps.202100349 34615368

[39] Perinelli E, Gremigni P. Use of social desirability scales in clinical psychology: a systematic review. J Clin Psychol 2016; 72: 534–51. 26970350 10.1002/jclp.22284

