# Supplementary material for: Destigmatising mental health treatment and increasing openness to seeking treatment: randomised controlled trial of brief video interventions
Source: BJPsych Open. 2022 Sep 16;8(5):e169. doi: 10.1192/bjo.2022.575 (PMC9534926; doi:10.1192/bjo.2022.575)
Supplement: Supplementary file 1 [file S2056472422005750sup001.docx]

**Supplemental**

**Appendix 1.** Links to the video interventions

1. Black female nurse - <https://youtu.be/Vkp7QHESKk4>
2. White female nurse - <https://youtu.be/myDJiqPrdSo>
